# Molecular Mechanisms of the SLC13A5 Gene Transcription

**DOI:** 10.3390/metabo11100706

**Published:** 2021-10-15

**Authors:** Zhihui Li, Hongbing Wang

**Affiliations:** Department of Pharmaceutical Sciences, University of Maryland School of Pharmacy, 20 Penn Street, Baltimore, MD 21201, USA; zli@rx.umaryland.edu

**Keywords:** SLC13A5, transcriptional regulation, xenobiotic receptor, pregnane X receptor, aryl hydrocarbon receptor

## Abstract

Citrate is a crucial energy sensor that plays a central role in cellular metabolic homeostasis. The solute carrier family 13 member 5 (SLC13A5), a sodium-coupled citrate transporter highly expressed in the mammalian liver with relatively low levels in the testis and brain, imports citrate from extracellular spaces into the cells. The perturbation of SLC13A5 expression and/or activity is associated with non-alcoholic fatty liver disease, obesity, insulin resistance, cell proliferation, and early infantile epileptic encephalopathy. SLC13A5 has been proposed as a promising therapeutic target for the treatment of these metabolic disorders. In the liver, the inductive expression of SLC13A5 has been linked to several xenobiotic receptors such as the pregnane X receptor and the aryl hydrocarbon receptor as well as certain hormonal and nutritional stimuli. Nevertheless, in comparison to the heightened interest in understanding the biological function and clinical relevance of SLC13A5, studies focusing on the regulatory mechanisms of SLC13A5 expression are relatively limited. In this review, we discuss the current advances in our understanding of the molecular mechanisms by which the expression of SLC13A5 is regulated. We expect this review will provide greater insights into the regulation of the *SLC13A5* gene transcription and the signaling pathways involved therein.

## 1. Introduction

**Figure 1:**
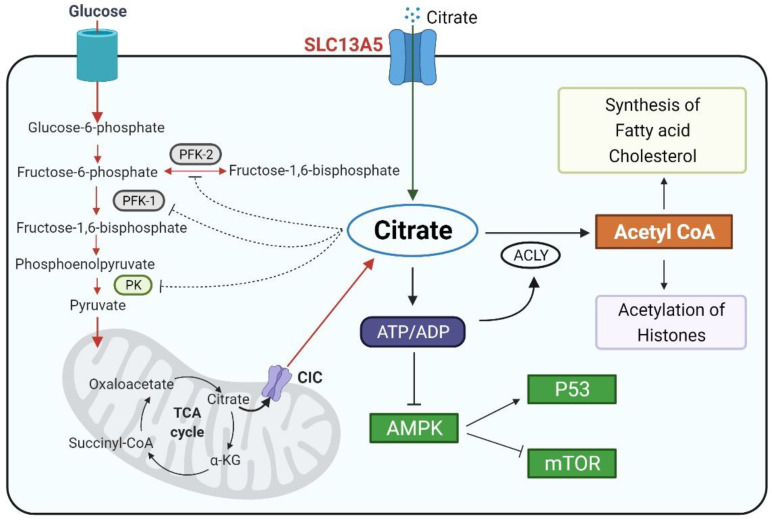
Schematic illustration of the cytosolic citrate balance and function. Citrate is synthesized in the mitochondria through the TCA cycle and transported to the cytosol via CIC (SLC25A1). Another source of cytosolic citrate is from extracellular spaces imported by SLC13A5/NaCT. Functionally, cytosolic citrate can (1) serve as a precursor for acetyl-CoA and play a key role in lipogenesis and histone acetylation; (2) suppress the glycolysis pathway via the inhibition of PFK1, PFK2, and PK, and (3) increase the ATP/ADP ratio and repress the AMPK signaling, affecting the cell proliferation. The schematic figures were created using BioRender (biorender.com, accessed on 4 October 2021).

The solute carrier family 13 member 5 (SLC13A5), also known as the Na^+^/citrate cotransporter (NaCT), is a member of the sodium dicarboxylate/sulfate cotransporter family. It recognizes and transports various dicarboxylate and tricarboxylate intermediates of the tricarboxylic acid (TCA) cycle with citrate as the preferred substrate [1,2]. Intracellular citrate functions as an energy sensor by influencing glycolysis, the TCA cycle, gluconeogenesis, and fatty acid synthesis, which are pivotal for the energy homeostasis of the cells [3,4]. The level of the cytosolic citrate is well-maintained by endogenous mitochondrial biosynthesis and exogenous uptake from the circulation. Mitochondrial citrate generated from the TCA cycle is transported outside the mitochondria by the citrate carrier (CIC), which is encoded by the *SLC25A1* gene [5]. Alternatively, cytosolic citrate can be imported from extracellular spaces via SLC13A5/NaCT expressed on the cell plasma membrane [6]. Cytosolic citrate is subsequently cleaved into acetyl-CoA and oxaloacetate by ATP citrate lyase (ACLY) [7]. Both acetyl-CoA and oxaloacetate are precursors of multiple anabolic reactions important for the de novo biosynthesis of fatty acids and steroids that are the building blocks for new cells [8]. On the other hand, elevated cytosolic citrate can repress glycolysis and the TCA cycle by inhibiting phosphofructokinase 1 (PFK1), PFK2, and pyruvate kinase (PK), key enzymes in the glycolysis pathway (Figure 1) [3,9]. In humans, the normal blood concentration of citrate is within the range of ~100–150 µM, which is several times higher than that of all other TCA cycle intermediates combined [10]. Thus, SLC13A5, being the primary uptake transporter of citrate, may play a key physiologic role in the generation of metabolic energy by facilitating the utilization of the circulating citrate. Specifically, in comparison to the ubiquitous biosynthesis of mitochondrial citrate and the broad expression of CIC/SLC25A1, SLC13A5 is preferentially expressed in tissues such as the liver, testis, and brain, which would influence physiological and pathophysiological conditions such as lipid metabolism, gluconeogenesis, obesity, type 2 diabetes, and epilepsy in an organ-specific pattern [1,11].

**Figure 2:**
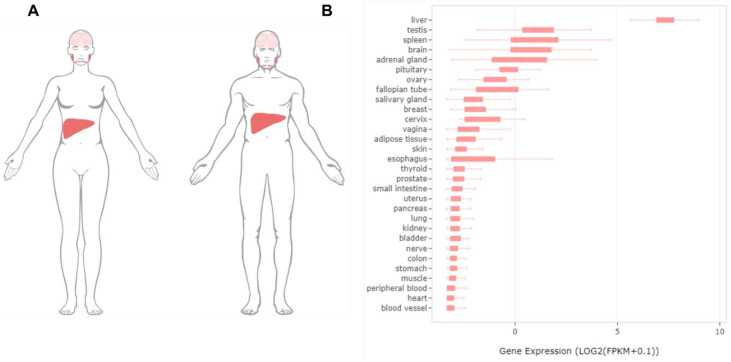
The distribution patterns of SLC13A5 in humans. (**A**) The global expression profiles of PXR in the human body (data available from https://www.proteinatlas.org/ENSG00000141485-SLC13A5/tissue#cbox, accessed on 14 July 2021). (**B**) The expression levels of SLC13A5 in different human tissues (generated via an Ingenuity Pathway Analysis (IPA); QIAGEN).

The cloning of the mammalian SLC13A5 was triggered directly by its structural and functional similarity with a Drosophila gene termed INDY, an acronym for “I’m Not Dead Yet”. The name of INDY was given by Rogina et al. based on the finding that the mutation of INDY was associated with a significant extension of the lifespan of fruit flies without affecting their energy intake [12]. Such a beneficial effect on the lifespan was also observed in *Caenorhabditis elegans* when the expression of an Na^+^-coupled citrate transporter (the C. elegans version of INDY) was repressed [13]. The subsequent structural-based functional postulation and experimental validation established INDY as a transporter protein that has a substrate preference for tricarboxylate citrate rather than for dicarboxylates [2]. SLC13A5/NaCT, the mammalian ortholog of Drosophila INDY (mINDY), was initially identified by screening a rat brain cDNA library with an EST (established sequence tag) probe, which led to the cloning of the full-length cDNA of the transporter as the newest member of the SLC13A family [2]. Subsequent studies revealed that, in humans, SLC13A5 mRNA is predominantly expressed in the liver followed by the testis, brain, spleen, bone marrow, and adrenal glands at much lower levels [14,15,16] (Figure 2). The human *SLC13A5* gene locates in chromosome 17 with a size of approximately 30 kb containing 12 exons, exhibiting a preferential uptake of tricarboxylate citrate that differs significantly from that of its sister transporters SLC13A2 (NaDC1) and SLC13A3 (NaDC3) [1,17]. In line with its predominance in the liver, a high expression of SLC13A5 has been found primarily localized in the sinusoidal membrane of hepatocytes, echoing its capacity of uptake citrate from the blood circulation [18,19]. Accumulating evidence has revealed that SLC13A5 plays an important role in controlling the hepatic citrate level and is linked to various metabolic disorders. The pharmacological inhibition and genetic silencing of SLC13A5 in the liver was reported to reduce hepatic lipid accumulation, improve hepatic insulin sensitivity, and repress liver tumor cell proliferation [20,21,22]. SLC13A5 knockout (−/−) mice were protected from high-fat diet (HFD)-induced obesity, a fatty liver, and insulin resistance [15,23]. On the other hand, the induction of SLC13A5 expressions in rat and human primary hepatocytes by benzo[a]pyrene (BaP) and rifampicin (RIF), respectively, resulted in an increased citrate uptake and/or hepatic lipid accumulation [24,25,26]. Notably, in addition to the perturbation of the SLC13A5 expression by xenobiotic and endobiotic chemicals, inherited genetic variants of SLC13A5 (particularly non-synonymous single nucleotide variants (SNVs) in the coding region) can directly affect the structure, activity, and expression of this transporter. To date, numerous naturally occurring mutations have been identified in the *SLC13A5* gene [27,28,29]. A few of these variants are associated with the disruption of the primary and secondary structure of the transporter, leading to protein internalization from the plasma membrane and a loss of the uptake function [29,30]. Most recently, human SLC13A5 protein structures in a complex with citrate or PF-06649298 (a known SLC13A5 inhibitor) were determined by Sauer et al., which provided structural insights for potential drug developments and the understanding of how various mutations affect the structure activity relationship of SLC13A5 [27].

Collectively, SLC13A5 has been established as a key transmembrane protein regulating cytosolic citrate levels in several energy-sensitive organs such as the liver and brain. The alteration of the SLC13A5 expression and function in these tissues is closely associated with obesity, type 2 diabetes, non-alcoholic fatty liver disease (NAFLD), inflammation, cancer, and neurological disorders. The purpose of this review is to highlight the recent progress in our understanding of the molecular mechanisms underlying the genetic and epigenetic regulation of the *SLC13A5* gene expression in a tissue-specific manner, with a specific focus centering on the hepatic regulation of the SLC13A5 transcription.

## 2. Upregulation of the SLC13A5 Expression

As an uptake transporter sensing intracellular citrate levels, the expression of SLC13A5 can be altered in response to various metabolic stresses and chemical challenges. An increased expression of SLC13A5 in the liver has been observed in patients with obesity, NAFLD, and type 2 diabetes as well as in cultured primary hepatocytes treated with glucagon, BaP, or RIF. Our understanding of the mechanisms underlying SLC13A5 upregulation has advanced significantly in the past ten years or so. 

### 2.1. PXR in the SLC13A5 Transcription

The pregnane X receptor (PXR, NR1I2), also known as the steroid X receptor (SXR), has been characterized as a master regulator of the expression of numerous phase I and phase II drug-metabolizing enzymes as well as drug transporters [31,32]. The perturbation of the PXR expression and activity can alter the absorption, distribution, metabolism, and excretion (ADME) profile of many drugs and results in clinically significant drug–drug interactions [33,34,35,36]. Hence, the screening of the PXR activity of drug candidates has been a common practice in numerous pharmaceutical companies. In addition to a xenobiotic disposition, mounting evidence suggests that PXR coordinates various physiological and pathophysiological processes involving energy homeostasis, inflammation, and cell proliferation [37,38,39,40,41,42,43]. Using wild-type, PXR knockout, and human PXR transgenic mice, several studies have revealed that the activation of PXR enhances lipogenesis whilst reducing lipid oxidation, resulting in a fatty liver phenotype [39,44,45]. In an attempt to decipher the transcriptional profile of the human PXR activation, a microarray analysis was conducted in human primary hepatocytes treated with RIF, the prototypical agonist of human PXR. Of note, *SLC13A5* was identified as one of the top genes that was robustly induced by RIF along with other known PXR target genes such as CYP3A4. This finding was subsequently confirmed by RT-PCR and Western blotting analyses in hepatocytes obtained from multiple human liver donors [25]. Notably, the RIF-mediated induction of SLC13A5 was markedly repressed by sulforaphane, a selective PXR deactivator [46], or the knockdown of the human PXR expression via lentiviral small hairpin RNA. Recently, the induction of the mRNA expression of a mouse Slc13a5 was also reported in mice treated with the prototypical mouse PXR activator, pregnenolone 16α-carbonitrile (PCN), albeit to a lesser extent [47].

Mechanistically, two clusters of potential PXR binding sites were identified at approximately −22 and −1.7 kb upstream of the SLC13A5 transcription start site, as shown in Figure 2. Utilizing cell-based luciferase reporter assays, electrophoretic mobility shift assays (EMSA), and chromatin immunoprecipitation (ChIP) assays, two distal enhancers containing the core site of AGGTCA spaced by 4 nucleotides (DR4) were functionally characterized as key elements involving the PXR-mediated transcription of the *SLC13A5* gene [25]. It is worth noting that whilst the induction of the *SLC13A5* gene by RIF in human primary hepatocytes is robust, the activation of the reporter construct of SLC13A5 containing the new DR4s by RIF is relatively moderate. Most recently, a comprehensive in silico analysis of the −30kb upstream and the intron regions of SLC13A5 was carried out to search for potential PXR response elements, which has led to the identification of two DR4s located in the introns of SLC13A5 with high raw scores (unpublished data). Although more functional experiments are expected, an initial EMSA analysis indicated that the new intron DR4s exhibit a potent binding capacity to the PXR/RXR heterodimer. Additionally, we noticed that the RIF-mediated activation of the SLC13A5 reporter gene in HepG2 cells cotransfected with PXR was markedly lower than that in human primary hepatocytes, which contained a full spectrum of liver-enriched transcription factors. Together, these findings suggest that additional novel response elements in the *SLC13A5* gene, as well as hepatic transcription factors other than PXR, might collectively contribute to the optimal induction of SLC13A5 in the liver. 

Phenobarbital (PB), although a prototypical activator of the constitutive androstane receptor (CAR, NR1I3) in rodents, is a dual activator of both human CAR and PXR, and can induce a large array of genes associated with drug metabolism and clearance [48,49]. Our recent results indicate that a PB treatment markedly induced the expression of SLC13A5 in both the mRNA and protein levels in human primary hepatocytes (unpublished data). Interestingly, the selective human CAR agonist, 6-(4-chlorophenyl)imidazo[2,1-b][1,3]thiazole-5-carbaldehyde-O-(3,4-dichlorobenzyl)oxime (CITCO), did not induce SLC13A5 expression either in human primary hepatocytes (unpublished data) or in HepaRG cells based on the analysis of our published RNA-seq data (GSE71446) [48]. Further studies using specific inhibitors of hCAR and hPXR as well as the HepaRG wild-type, CAR−/−, and PXR−/− cell lines revealed that PB induces the expression of SLC13A5 in liver cells via a PXR-mediated signaling pathway independent of CAR (unpublished data). Collectively, these findings indicate that although PXR and CAR share many target genes that encode drug-metabolizing enzymes and drug transporters, the transcription of SLC13A5 is upregulated by the activation of PXR but not CAR.

### 2.2. AhR in the SLC13A5 Transcription

**Figure 3:**
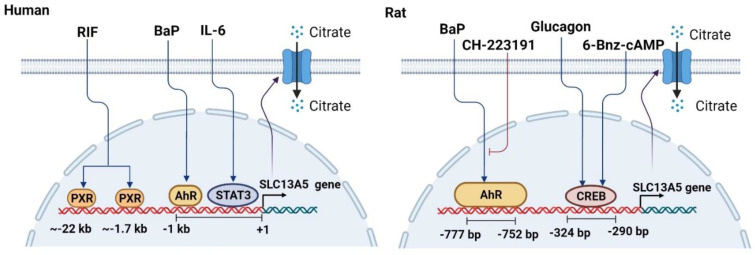
Schematic illustration of the mechanisms underlying the transcriptional regulation of SLC13A5. Transcription factors including PXR, AhR, CREB, and STAT3 can positively regulate the transcription of SLC13A5 in the liver upon exposure to their activators such as RIF, BaP, glucagon, and IL-6, respectively. Specific response elements (motifs) that can bind to these transcription factors have been identified upstream of the *SLC13A5* gene in human and/or rodents. CH-223191 is an antagonist of AhR.

The aryl hydrocarbon receptor (AhR) is a ligand-activated transcription factor that belongs to the basic helix-loop-helix PER-ARNT-SIM (PAS) gene family [50]. Inactivated AhR is expressed primarily in the cytoplasm as a protein complex with several chaperones including two heat shock protein 90 (HSP90), a co-chaperone p23, and a XAP-2 molecule [51,52]. The activation of AhR involves ligand binding, nuclear translocation, and interaction with the promoter of the target genes after forming a heterodimer with its partner, AhR nuclear translocator (ARNT) [36,53]. As with PXR and CAR, AhR has been referred to as an important xenobiotic sensor dictating the inductive expression of many drug-metabolizing enzymes and transporters including CYP1A1, CYP1A2, CYP1B1, UGT1A1, UGT1A3, UGT1A4, UGT1A6, and BCRP [54,55,56,57,58,59,60]. The key biological functions of AhR also include the regulation of energy metabolism and immune/inflammatory responses [61,62]. The activation of AhR was reported to induce spontaneous hepatic steatosis characterized by the accumulation of triglycerides likely via the upregulation of fatty acid uptake transporters whilst suppressing fatty acid oxidation and export [63]. A strong correlation between an exposure to AhR-activating chemicals such as polychlorinated biphenyls (PCBs) and increased metabolic disorders has been experimentally established [64,65]. At around the same time that PXR was recognized as a modulator of SLC13A5 expression, Neuschafer-Rube et al. reported that the mRNA expression of SLC13A5 was induced by a number of AhR activators including BaP, 2,3,7,8-tetrachlorodibenzodioxin (TCDD), and 3-methylcholanthrene (3MC) in cultured rat primary hepatocytes with BaP demonstrating the most robust induction (8-fold) [24]. The BaP-mediated induction of SLC13A5 was markedly attenuated by CH223191, a known AhR antagonist. A putative AhR binding site in the promoter of SLC13A5 was subsequently identified and evaluated as important for AhR/ARNT binding and the transactivation of the *SLC13A5* gene (Figure 3). Functionally, the activation of AhR signaling by BaP increased the hepatic citrate uptake and resulted in an increased incorporation of radioactively-labelled citrate into lipids [24].

It is worth mentioning that the magnitude of SLC13A5 induction in rat primary hepatocytes by BaP (8-fold) at 20 µM is significantly higher than the approximately 2-fold increase achieved by the treatment of either TCDD (10 nM) or 3MC (1 µM), two prototypical AhR agonists. It is well-known that TCDD and 3MC at the concentrations of 10 nM and 1 µM, respectively, elicit a robust activation of AhR and the induction of its prototypical target genes CYP1A1 and CYP1A2 [66,67]. The obviously moderate induction of SLC13A5 achieved by TCDD and 3MC in comparison to BaP suggests the potential involvement of molecular mechanisms other than AhR. Indeed, additional experiments demonstrated a dose-dependent induction of SLC13A5 by BaP through concentrations up to 100 µM. Higher concentrations of BaP at and above 20 µM have previously been indicated to be toxic to hepatocytes [68,69]. Whether BaP causes cellular toxic responses and also contributes to its upregulation of SLC13A5 remains elusive. 

### 2.3. CREB in the SLC13A5 Transcription

The cAMP-responsive element-binding protein (CREB) is a member of the basic leucine zipper (bZIP) class of transcription factors, regulating a variety of genes associated with glucose sensing, lipid metabolism, and fibrosis in response to the elevated second messenger cAMP [70]. Phosphorylated CREB binds to the cAMP response element (CRE) in the promoter region of its target genes, provoking the glucagon-PKA-cAMP axis as one of the primary CREB-activating signaling cascades [71].

In addition to a xenobiotic stimulation, an endogenous hormonal and/or nutritional status has been linked to the expression of SLC13A5 in mammals. In a cohort of human liver tissue samples, von Loeffelholz et al. found that the hepatic expression of SLC13A5 was positively associated with body mass index (BMI), waist circumference, body fat, and a histologically assessed liver fat content [26]. An experimental perturbation of SLC13A5 expression was also observed in mice fed with a HFD or under fasting conditions. In rat primary hepatocytes, the physiological concentration of glucagon (10 nM) significantly induced the mRNA expression of SLC13A5 and increased the hepatic uptake of [^14^C]-citrate [72]. It is well-known that glucagon interacts with its cell-surface receptor to increase the intracellular cAMP levels, which can in turn phosphorylate and activate the transcription factor CREB [73]. Subsequent mechanistic studies uncovered a functional CRE core site located near -324 in the promoter of SLC13A5; the mutation of the core sequence disrupted the CREB binding and abolished the glucagon-dependent induction of the SLC13A5 luciferase reporter activity. Notably, the expression of SLC13A5 was also induced in the liver of overnight-fasting and type 2 diabetic rats where both conditions elevated the CREB activity [72,74]. Of clinical importance, these hormonally and/or nutritionally enhanced uptakes of citrate via SLC13A5 upregulation can be converted by ACLY to acetyl-CoA and oxaloacetate, critical steps influencing lipogenesis and gluconeogenesis in the liver (Figure 1). Glucagon treatment in rat primary hepatocytes increased the incorporation of [^14^C]-labelled citrate into newly synthesized glucose [72].

Recently, Kopel et al. found that the anti-diabetic drug metformin suppresses the expression of SLC13A5 in HepG2 cells [75], albeit at a concentration (10 mM) that is several magnitudes higher than its pharmacological level (~40 µM) [76]. The signaling pathway for the metformin-mediated repression of the *SLC13A5* gene is linked to its activation of AMP-activated protein kinase (AMPK), which in turn inhibits the mammalian target of rapamycin complex 1 (mTORC1) and CREB. The involvement of the AMPK pathway was further evidenced with a similar observation when the cells were treated with AICAR, another AMPK activator. Together, these results suggest that the activation of CREB may represent a common mechanism shared by both hormonal and nutritional stimuli in the upregulation of a hepatic SLC13A5 expression.

### 2.4. STAT3 in the SLC13A5 Expression

Cytosolic citrate is a central metabolite at the crossroads of energy homeostasis, connecting carbohydrate catabolism, lipogenesis, and gluconeogenesis, which has recently been shown to be enhanced by interleukin-6 (IL-6) [77]. IL-6 is a multifunctional cytokine that not only regulates immune and inflammatory responses but also affects hematopoiesis, metabolism, and organ development [78]. It is known that IL-6 levels are increased in obesity, type 2 diabetes, NAFLD, and several metabolic states that are associated with an elevated SLC13A5 expression [26,79,80,81]. 

Combining in vitro cell cultures with in vivo genetically modified mouse models and human patient samples, von Loeffelholz et al. provided experimental evidence demonstrating SLC13A5 as a target gene of a signal transducer and activator of transcription 3 (STAT3) in response to IL-6 stimulation [26]. Both the treatment of human primary hepatocytes with IL-6 and the intravenous administration of IL-6 in mice markedly increased the hepatic SLC13A5 mRNA levels. In contrast, blocking the IL-6 receptor (IL-6R) using a monoclonal antibody (tocilizumab) or through a mouse liver-specific IL-6R knockout model abolished the IL-6-mediated induction of SLC13A5. Such a correlation was also observed in NAFLD patients where SLC13A5 expression was 2-fold higher in patients with plasmid IL-6 levels above 4.81 pg/mL compared with patients with IL-6 below this cutoff line. Subsequently, two responsive elements containing the consensus sequence TT(N4-6)AA of STAT3 were identified close to −376 bp and −618 bp in the promoter of SLC13A5. IL-6 treatment efficiently activated the luciferase reporter constructs containing these two STAT3 binding sites. Nevertheless, the site-directed mutation of the STAT3 binding sites did not repress the luciferase activity stimulated by IL-6. Given the lack of evidence in this article to show that STAT3 can bind to these two responsive elements, it is possible that response elements other than these two may contribute to the observed activation. Clearly, more in-depth investigations are needed to define the mechanisms of the IL-6-mediated induction of SLC13A5.

## 3. Downregulation of the SLC13A5 Expression

The clinical significance of a decreasing SLC13A5 expression and activity is evident. However, many studies were conducted by artificially manipulating the SLC13A5 expression/activity via a gene knockdown, knockout, or chemical inhibition. In comparison with the upregulation of SLC13A5, much less is known regarding the mechanisms underlying its downregulation. The current literature suggests that genetic polymorphisms and the epigenetic modification of the *SLC13A5* gene may contribute to its observed low expression in certain individuals. 

### 3.1. Naturally Occurring Mutations of the SLC13A5 Gene

**Figure 4:**
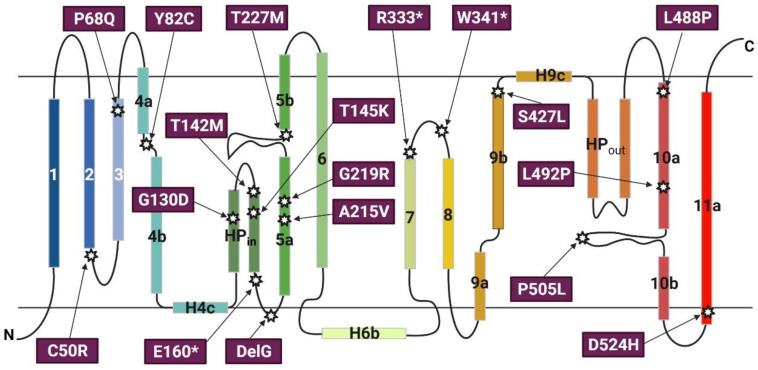
Transmembrane topology of SLC13A5 and the localization of selected non-synonymous single nucleotide variants (SNVs). The transmembrane topology structure of SLC13A5 was adopted from Sauer et al. The 11 transmembrane helices are shown as numbered rectangles; the hairpin loop is labelled HP. The localization and amino acid replacement of selected SNVs previously reported as “pathogenic” are indicated. * Represents nonsense mutations.

The effects of genetic variants on gene expressions and associated disease propensity have long been recognized. Accumulating evidence reveals that several non-synonymous mutations of the *SLC13A5* gene are phenotypically linked to neonatal epilepsy, developmental delay, and tooth hypoplasia [28,30,82]. Early onset epileptic encephalopathy (EOEE) is a clinically and etiologically heterogeneous subgroup of epilepsy syndromes with a developmental delay and high mortality rate [28]. A genetic analysis on three families with an autosomal-recessive inheritance pattern of epileptic encephalopathy by Thevenon et al. resulted in the identification of three missense SNVs, c.680C > T [p.Thr227Met], c.655G > A [p.Gly219Arg], and c.1463T > C [p.Leu488Pro] that are associated with patients who have a seizure onset in the early days of life [83]. Subsequent studies have discovered more than 40 SNVs in the coding region of SLC13A5 that were termed “pathogenic SNVs”, as exemplified in Figure 4, contributing to the development of EOEE, tooth hypoplasia, and osteogenesis imperfecta [27,84]. 

Functionally, these variants in the coding region of the *SLC13A5* gene often cause a decrease or loss of the citrate uptake capacity as the result of (1) an incompletely folded or unassembled protein that is internalized from the plasma membrane, or (2) the mutated protein has a defected binding pocket preventing sodium and/or citrate from interacting with it [28]. Of importance, because energy production in the brain is heavily reliant on the citric acid cycle, the loss of SLC13A5 function/expression is expected to have a profound influence on the synthesis of neurotransmitters such as acetylcholine, glutamate, and GABA in the central nervous system.

Compared with the heightened focus on studying missense SNVs of SLC13A5, the investigation of genetic variants occurring in the non-coding regions of SLC13A5 (the upstream/downstream of 5′ and 3′ untranslated regions as well as introns) has not been reported to date. A bioinformatic analysis of the SLC13A5 mutations located in the non-coding regions from the Exome Aggregation Consortium (ExAC) dataset (https://gnomad.broadinstitute.org, accessed on 3 July 2021) revealed a total of 420 variants in the non-coding regions with 15 reaching a frequency > 1% in the population (Table 1). However, the phenotypical significance of these variants is unknown. Theoretically, variants in the non-coding regions, particularly in the promoter regions, are expected to influence the overall expression of the gene without affecting its protein structure [85,86,87]. Thus, it is highly likely that concurrent mutations in both the coding and non-coding regions of SLC13A5 may contribute cooperatively to the development of deleterious neurological phenotypes.

### 3.2. Epigenetic Regulation of SLC13A5

In addition to the genetic control of the gene regulation, epigenetic modifications can alter the DNA accessibility and chromatin structure, thereby regulating the gene expression and playing a pivotal role in the normal and pathophysiological development and differentiation [89]. Major epigenetic mechanisms include DNA methylation, histone modification, and regulatory non-coding RNAs such as microRNAs and circular RNAs [90]. 

DNA methylation at the C5 position of cytosine in CpG dinucleotides represents one of the central epigenetic mechanisms that suppresses the gene expression. A whole-genome integrative analysis of methylation and the gene expression profiles in glioblastoma and normal brain tissues reveals that SLC13A5 is one of the 13 genes with concordant CpG islands in their promoter regions and the hypermethylation of the CpG sites of SLC13A5 is inversely correlated to its low expression in glioblastomas [91]. In a separate report, Diaz et al. found that the *SLC13A5* gene was hypermethylated and downregulated in the placenta of individuals born small-for-gestational-age (SGA) who have less adipose tissue and more insulin sensitivity than appropriate-for-gestational-age (AGA) infants [92]. Similar results were also observed in renal cancer cells where DNA hypermethylation on the CpG islands of the *SLC13A5* gene was identified in a cluster of renal cancer patients with poor survival rates [93]. Together, these findings suggest that with multiple CpG islands located in the promoter region of the *SLC13A5* gene, its expression is sensitive to the type of DNA methylation and epigenetic modulations. Tissue-specific DNA hypermethylation resulting in a low expression of SLC13A5 may be an additional molecular mechanism contributing to inheritable neurological and developmental disorders. 

## 4. Conclusions and Perspectives

As a crucial energy sensor regulating cytosolic citrate levels, SLC13A5 plays crucial metabolic roles maintaining energy homeostasis in the liver, brain, and several other tissues. An abnormal expression and/or activity of SLC13A5 is associated with hepatic metabolic disorders, severe early onset epilepsy, and several other disease conditions. In the past decade, significant progress has been achieved in our understanding of the molecular mechanisms by which the expression of SLC13A5 is regulated. As summarized in Table 2, transcription factors including PXR, AhR, CREB, and STAT3 have been identified as positive regulators that can upregulate SLC13A5 transcription upon xenobiotic or endobiotic stimulation. Notably, these findings are based primarily on studies conducted using hepatocyte cultures in vitro or in the liver of rodents. Given that regulation of the gene expression is generally considered to be a tissue-specific event, whether SLC13A5 in extrahepatic tissues such as the brain would be regulated in the same manner is largely unknown. Thus, the identification of the tissue-specific inducers of SLC13A5, particularly in the brain and bone marrow, may hold a greater clinical importance. In contrast to upregulation, our knowledge regarding the repression of SLC13A5 transcription is limited. Intriguingly, the depletion of SLC13A5 in the liver has demonstrated several metabolic benefits. For instance, we have shown that the silencing of SLC13A5 expression leads to a reduction in the proliferation of HepG2 and Huh7 cells accompanied by decreases in de novo lipogenesis through the activation of the AMPK-mTOR signaling pathway [20]. Moreover, the knockdown of SLC13A5 promotes hepatic ketogenesis and cellular stress, which sensitizes the response of HepG2 cells to chemotherapeutic agents [94]. Thus, understanding the mechanisms for SLC13A5 downregulation and developing potent and selective SLC13A5 modulators warrants future investigation.

Several loss-of-function mutations of the *SLC13A5* gene have been closely linked to the development of EOEE. Although these missense SNVs in the coding region are deemed to affect protein conformation and function, several studies have indicated a reduction of SLC13A5 expression in a few of these patients. Whether concurrent SNVs located in the non-coding region of SLC13A5 co-contribute to neural disorders remains to be investigated. It is evident now that the perturbation of the SLC13A5 expression and activity in the central nervous system vs. in the liver may have opposite clinical consequences. Although enhancing the SLC13A5 function in the brain is expected to benefit EOEE patients, repressing its hepatic expression and activity may represent a therapeutic strategy for several metabolic disorders. Therefore, an improved understanding of the signaling control of SLC13A5 transcription in a tissue-specific manner will eventually benefit the development of SLC13A5 modulators as potential drug candidates for SLC13A5-associated diseases in humans.

## Figures and Tables

**Table 1:** The variants of the *SLC13A5* gene in the non-coding region (allele frequency > 1%) ^a^.

Chromosome	Position	rsIDs ^b^	Reference	Alternate	Annotation	Allele Frequency (%)
17	6616669	rs62061545	T	G	5_prime_UTR_variant	2.063
17	6616527	rs62621831	G	A	intron_variant	8.467
17	6610536	rs62061541	A	G	intron_variant	1.730
17	6610295	rs144316437	AAG	A	intron_variant	4.250
17	6607435	rs218678	G	A	intron_variant	9.240
17	6607389	rs218677	G	A	intron_variant	2.729
17	6607150	rs62061540	G	A	intron_variant	2.400
17	6599288	rs77795940	C	T	intron_variant	3.760
17	6596497	rs896810583	A	AAC	intron_variant	8.085
17	6594322	rs4796540	G	A	intron_variant	23.338
17	6594291	rs116929585	C	T	intron_variant	1.502
17	6593417	rs218694	A	G	intron_variant	12.780
17	6593414	rs934718226	AC	A	intron_variant	5.223
17	6591041	rs78203589	C	T	intron_variant	1.335
17	6589495	rs16956120	A	G	3_prime_UTR_variant	1.073

^a^ Gene location based on the National Center for Biotechnology Information (NCBI) Human Genome Assembly Build GRCh37/hg19. The data were collected from https://gnomad.broadinstitute.org, accessed on 3 July 2021 [88]. ^b^ Reference SNP cluster ID.

**Table 2:** Transcription factors or xenobiotic chemicals that regulate *SLC13A5* gene expression.

		Species	Tissue	Effect ^a^	Reference
	PXR	Human	Human primary hepatocytes	+	[25]
	AhR	Rat	Rat primary hepatocytes	+	[24]
**Transcription Factor**		Mouse	Mouse primary hepatocytes	+	
	CREB	Rat	Rat primary hepatocytes	+	[72]
	STAT3	Human	Human primary hepatocytes	+	[26]
		
	Glucagon	Rat	Rat primary hepatocytes	+	[72]
**Hormone or Cytokine**	IL-6	Human	Human primary hepatocytes	+	[26]
		Mouse	Mouse liver	+	
	RIF	Human	Human primary hepatocytes	+	[25]
	TCDD	Rat	Rat primary hepatocytes	+	[24]
	3MC	Rat	Rat primary hepatocytes	+	[24]
	BaP	Rat	Rat primary hepatocytes	+	[24]
		Mouse	Mouse primary hepatocytes	+	
**Chemicals or Others**	Metformin	Human	HepG2 cells	−	[75]
	AICAR	Human	HepG2 cells	−	[75]
	Phenobarbital	Rat	Rat primary hepatocytes	+	[24]
	Bisphenol-A	Mouse	Mouse liver	−	[95]
	CTPI-2	Mouse	Mouse liver	+	[96]
	LPS	Human	Human non-parenchymal cells (including Kupffer cells) were co-cultivated with human primary hepatocytes	+	[26]
	6-Bnz-cAMP	Rat	Rat primary hepatocytes	+	[72]

^a^ +, induction of the *SLC13A5* gene; −, repression of the *SLC13A5* gene.
